# Prevalence of antimicrobial resistance in acute community-acquired urinary tract infections in Sub-Saharan Africa: a systematic review

**DOI:** 10.1093/jacamr/dlag155

**Published:** 2026-08-17

**Authors:** Amelia Williams-Walker, Lucy Lammie, Catrin E Moore

**Affiliations:** Institute of Infection and Immunity, School of Health and Medical Sciences, Department of Medicine, City St George’s, University of London, London, UK; Institute of Infection and Immunity, School of Health and Medical Sciences, Department of Medicine, City St George’s, University of London, London, UK; Institute of Infection and Immunity, School of Health and Medical Sciences, Department of Medicine, City St George’s, University of London, London, UK

## Abstract

**Background:**

In Sub-Saharan Africa, over-the-counter antibiotic dispensing and limited resources drive high antimicrobial resistance (AMR), yet data on community-acquired urinary tract infections (UTIs) remain scarce. This review synthesizes available AMR evidence in this population.

**Methods:**

We searched MEDLINE, Embase and Global Health (January 2000–April 2026) for studies evaluating AMR in acute community-acquired UTIs across Sub-Saharan Africa. Random-effects meta-analyses evaluated resistance of *Escherichia coli* and *Klebsiella* spp. against five commonly prescribed antibiotics.

**Results:**

From 3099 screened articles, 42 studies (19 countries) were analysed. Pooled AMR prevalence was high for *E. coli* and *Klebsiella* spp., respectively: trimethoprim-sulfamethoxazole [62.2% (95% CI: 51.9%–72.5%) versus 66.4% (95% CI: 46.7%–86.2%)]; amoxicillin-clavulanic acid [48.9% (95% CI: 39.8%–58.0%) versus 47.5% (95% CI: 31.0%–63.2%)]; ceftriaxone [40.8% (95% CI: 19.4%–62.2%) versus 44.6% (95% CI: 28.6%–60.7%)]; ciprofloxacin [32.8% (95% CI: 25.1%–40.4%) versus 35.8% (95% CI: 20.3%–51.4%)] and nitrofurantoin [18.2% (95% CI: 9.2%–27.2%) versus 28.3% (95% CI: 12.1%–44.5%)]. All estimates showed very high heterogeneity (*I*^2^ > 96%), unresolved by subgroup analysis. *E. coli* resistance to amoxicillin-clavulanic acid increased significantly over time (*P* = 0.028). Resistance did not differ significantly by pathogen or geographic region.

**Conclusions:**

Community-acquired UTIs in Sub-Saharan Africa exhibit high rates of AMR. Addressing this burden requires targeted community antimicrobial stewardship, rapid diagnostic tools to guide empiric therapy and standardized laboratory reporting to enable accurate global surveillance.

## Introduction

Urinary tract infections (UTIs) are a significant global public health burden as they are one of the most prevalent bacterial infections occurring in the community, with an estimated 449 million incidents annually.^1–3^ Treating UTIs cost National Health Service hospitals in the UK >£600 million from 2023 to 2024, however, it is far more difficult to quantify this cost in the community.^4^ Clinically, a UTI is characterized by a significant number of bacteria in the bladder with symptoms arising from the pathogenic inflammation of the urinary tract.^5^ While the microbial aetiology of UTIs is diverse and the methodology often discussed, *Escherichia coli (E. coli*) is the most frequent uropathogen responsible for >75% of acute UTIs.^6^ Other frequently diagnosed causative agents include *Klebsiella pneumoniae*, *Staphylococcus saprophyticus* and *Enterococcus* species.^7^

Acute cystitis, defined as an infection that is localized to the lower urinary tract and bladder, is the most common clinical UTI presentation.^5^ Mild cases are usually managed through non-therapeutic interventions such as increasing fluid intake; pharmacological interventions (e.g. antibiotics) remain the standard of care for patients with persistent or severe symptoms. The efficacy of these treatments is threatened by the global escalation of antimicrobial resistance (AMR).^8^

Community-acquired UTIs (CA-UTIs) are often treated with empiric antibiotics, which is driven in part by a global deficit in rapid, point-of-care urinary diagnostic tests for use outside of microbiology departments.^9^ Even though care relies on empiric antibiotic use, many countries in low- and middle-income countries such as Sub-Saharan Africa do not have up-to-date standard treatment guidelines (STGs) to treat UTIs, this recently led to the Africa Centres for Disease Control and Prevention (CDC) publishing a continent-wide STG to advise the management of common infectious diseases in 2021.^10^ These guidelines, such as the World Health Organization (WHO) AWaRe (Access, Watch, Reserve) Antibiotic book, prioritize the use of nitrofurantoin or amoxicillin-clavulanic acid as first-line treatments for uncomplicated UTIs with ciprofloxacin reserved as an alternative.^11^ Despite high local resistance towards trimethoprim-sulfamethoxazole because of its use for other infectious diseases across Africa, some national guidelines still recommend it for use in UTI treatment, highlighting the importance of local burden knowledge and the need to update guidelines regularly.^12–14^ There is a lack of consensus on the definition of complicated UTIs but nevertheless ceftriaxone or gentamicin are often the recommended treatment options.^10,15^ The WHO AWaRE guidelines recommend the five antibiotics chosen for analysis in this review (nitrofurantoin, amoxicillin-clavulanic acid, trimethoprim-sulfamethoxazole, ceftriaxone and ciprofloxacin) as first-choice treatments for lower UTIs and mild pyelonephritis.^11^

Many parts of Sub-Saharan Africa suffer from a lack of laboratory diagnostic capabilities; this is even more pronounced in the community as highlighted by the Mapping Antimicrobial Resistance and Antimicrobial Use Partnership project between 2016 and 2019. They surveyed 53 770 laboratories across 14 African countries and found only 1% of medical laboratories able to conduct bacteriology testing and 393 laboratories (0.73%) able to perform antibiotic susceptibility testing, thus highlighting this important diagnostic gap.^16–18^ Furthermore, a lack of access to antibiotics in some public facilities contrasted by the widespread availability of antibiotics through informal (both public and private) sectors which contributes to antibiotic misuse/overuse.^19–21^

Therefore, the antimicrobial resistance (AMR) prevalence of CA-UTIs in Sub-Saharan Africa remains poorly characterized. This systematic review aims to synthesize the available AMR data of community UTI strains (*E. coli* and *Klebsiella* spp.) to address this gap. In addition, this review aims to understand whether there are geographical or temporal variations in resistance rates for CA-UTIs in this region.

## Methods

This systematic review adhered to the Preferred Reporting Items for Systematic Reviews and Meta-Analyses (PRISMA) guidelines.^22^ The protocol was registered on the International Prospective Register of Systematic Reviews (CRD42024611890).

### Search strategy

A systematic search of peer-reviewed literature (all languages) published between January 2000 and April 2026 was performed using MEDLINE, Embase and Global Health databases. The comprehensive search strategy included an exhaustive list of terms for ‘urinary tract infection’, ‘antimicrobial resistance’ and ‘community’. Each country within the World Bank definition of Sub-Saharan Africa was listed individually (including historical names for certain territories) along with other key terms for this region and the search terms were combined using Boolean operators (Table S1, available as Supplementary data at *JAC-AMR* Online).^23^

### Selection criteria

Studies were screened independently at the title and abstract stage on RefWorks by two reviewers (A.W.W. and L.L.). The third reviewer (C.M.) was consulted in the case of any disagreements.

Clear inclusion and exclusion criteria were established for this review to ensure only articles that presented data on AMR in acute community-acquired UTIs in Sub-Saharan Africa were selected for analysis. Articles published between 1 January 2000 and 1 April 2026 were eligible for inclusion; this period was chosen to ensure the collection of spatial and temporal trends together with clinically relevant resistance patterns.

Included studies containing quantitative information on patients of any age diagnosed with an acute community-onset UTI with phenotypic antimicrobial susceptibility testing (AST) results were included. This review only assessed studies that specified their study location as Sub-Saharan Africa. Studies with AST results for fewer than 10 cultured isolates were excluded, along with studies where patients had comorbidities (such as diabetes, HIV, individuals with urinary tract abnormalities or those known to be pregnant) where the UTI could be classified as complicated (or asymptomatic disease in the case of pregnancy; Table S2). This exclusion criteria aimed to improve comparability between studies and reduce the impact that the distinct pathophysiology and altered AMR profiles associated with these different presentations might have on the overall analysis.

Both prospective and retrospective studies were eligible for inclusion if they included stratified data on community-acquired UTIs (e.g. if the study location was specified as the outpatient department of a hospital).

### Data extraction

Data from included studies were extracted onto a standardized Excel spreadsheet. All studies were extracted by two independent researchers (A.W.W. and L.L.) and reviewed for any inconsistencies. The following variables were extracted: study start and end date, country, year of publication, study setting, UTI culture definition, number of enrolled patients, number of positive UTI patients, number of positive isolates, organism species, AST methodology, AST guidelines, internal quality controls and AST results. If any of the listed variables were not included in the studies, this was noted during data extraction.

### Quality assessment

We determined the risk of bias using an adapted descriptive tool. This included a scale from 1 to 4 based on methodological controls and data completeness. The three factors that were evaluated included the specification of a UTI-positivity cut-off, AST guidelines and internal quality controls alongside any missing or inconsistent data for each study (Table S2). However, no studies were excluded from the analysis on the basis of these factors due to a lack of consensus on standardized reporting in microbiological studies.

### Data analysis

The sample collection midpoint was calculated for each study, if this was not available, it was inferred using the median time difference between all study midpoints and their publication dates. The number of susceptible and intermediate isolates from each study were grouped and designated as susceptible following the 2019 European Committee on Antimicrobial Susceptibility testing (EUCAST) guidelines.^24^

Individual studies were combined using random-effect meta-analysis, which best accounts for the between-study heterogeneity that was seen. Forest plots were created to show the resistance rates of the two most common UTI pathogens (*E. coli* and *Klebsiella* sp.) against the most clinically relevant antibiotics for UTI treatment (amoxicillin-clavulanic acid, ceftriaxone, ciprofloxacin, nitrofurantoin and trimethoprim-sulfamethoxazole). Studies were grouped into 5-year time blocks (e.g. 2000–2004, 2005–2009, 2010–2014, 2015–2019, 2020–2025) on the basis of the study period. This 5-year interval was chosen to ensure a balance between temporal resolution and to provide sufficient statistical power within each subgroup. These blocks were used to compare how resistance changed across Sub-Saharan Africa over time, comparing each time block to the previous one. Heterogeneity between the studies was examined using forest plots and the *I*^2^ statistic. Summary statistics were created for each time block per organism and antibiotic combination with 95% confidence intervals. Subgroup analysis was conducted when there were sufficient studies in each subgroup (≥3), for geographical region, temporal group and quality score. All meta-analysis and forest plot creation was performed in the R package metafor (v.4.8.0). Statistical tests were performed using R packages emmeans (v.1.11.1) and lme4 (v.1.1–37). *Z*-tests with Bonferroni correction were used to determine statistically significant differences in resistance prevalence between organisms or antibiotics. Publication bias was assessed for each organism-antibiotic combination with >10 studies, using an asymmetric funnel plot and Egger’s test.^25^

## Results

**Figure 1.**
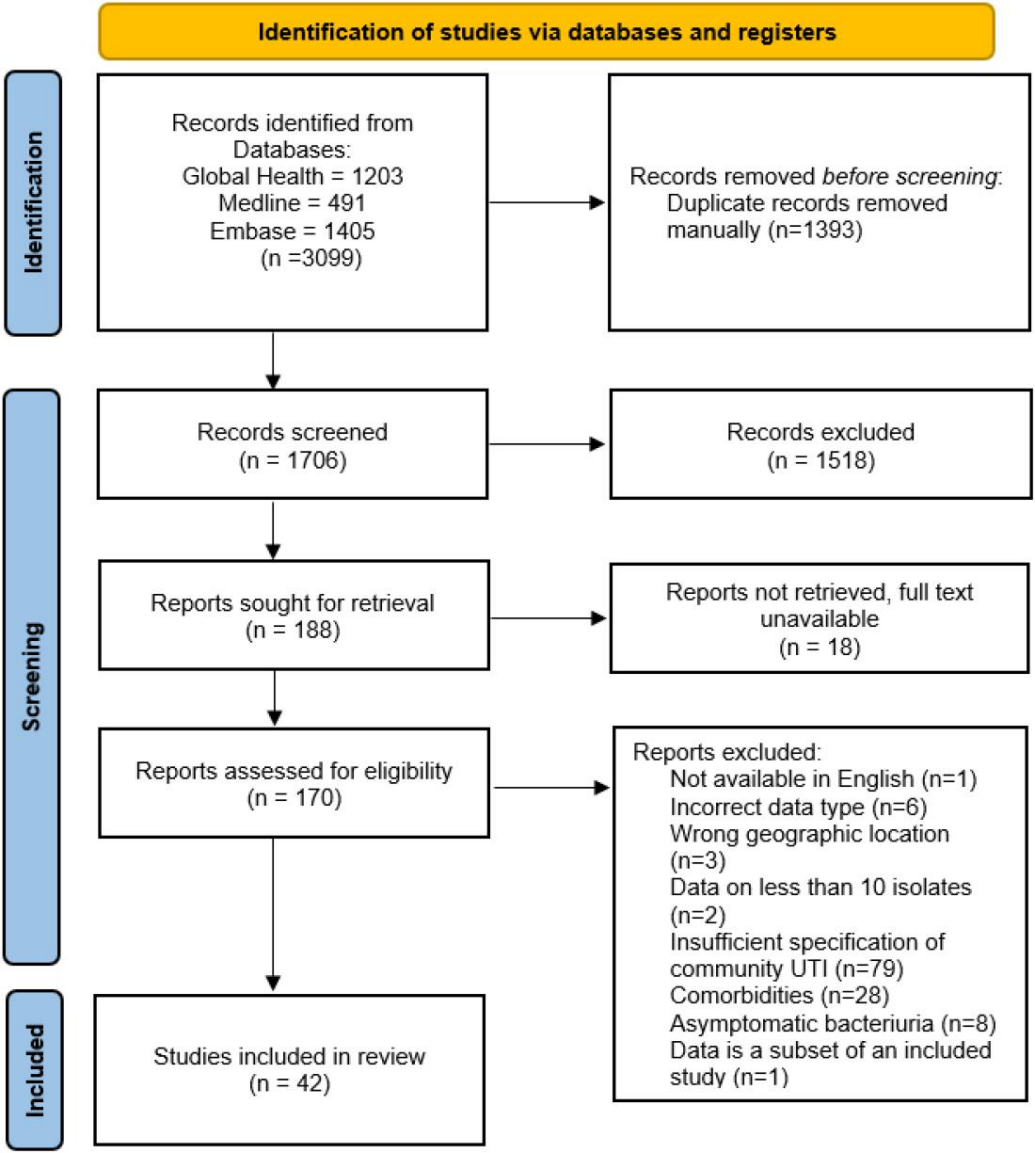
PRISMA flow diagram showing the search results and reasoning behind paper exclusion.

The systematic database search identified 3099 articles; 1518 were excluded during abstract screening due to not meeting the inclusion criteria. Full texts were not available for 18 articles, and 128 were excluded at the full-text screening stage mainly due to inadequate separation between hospital and community infections (Figure 1). Forty-two studies met the inclusion criteria, one study (Holistic Approach to Unravel Antibacterial Resistance in East Africa (HATUA)) was described in two separate eligible reports; this was treated as a single unit of analysis to prevent data duplication (Table S3).^17,26^

### Descriptive summary of studies

**Figure 2.**
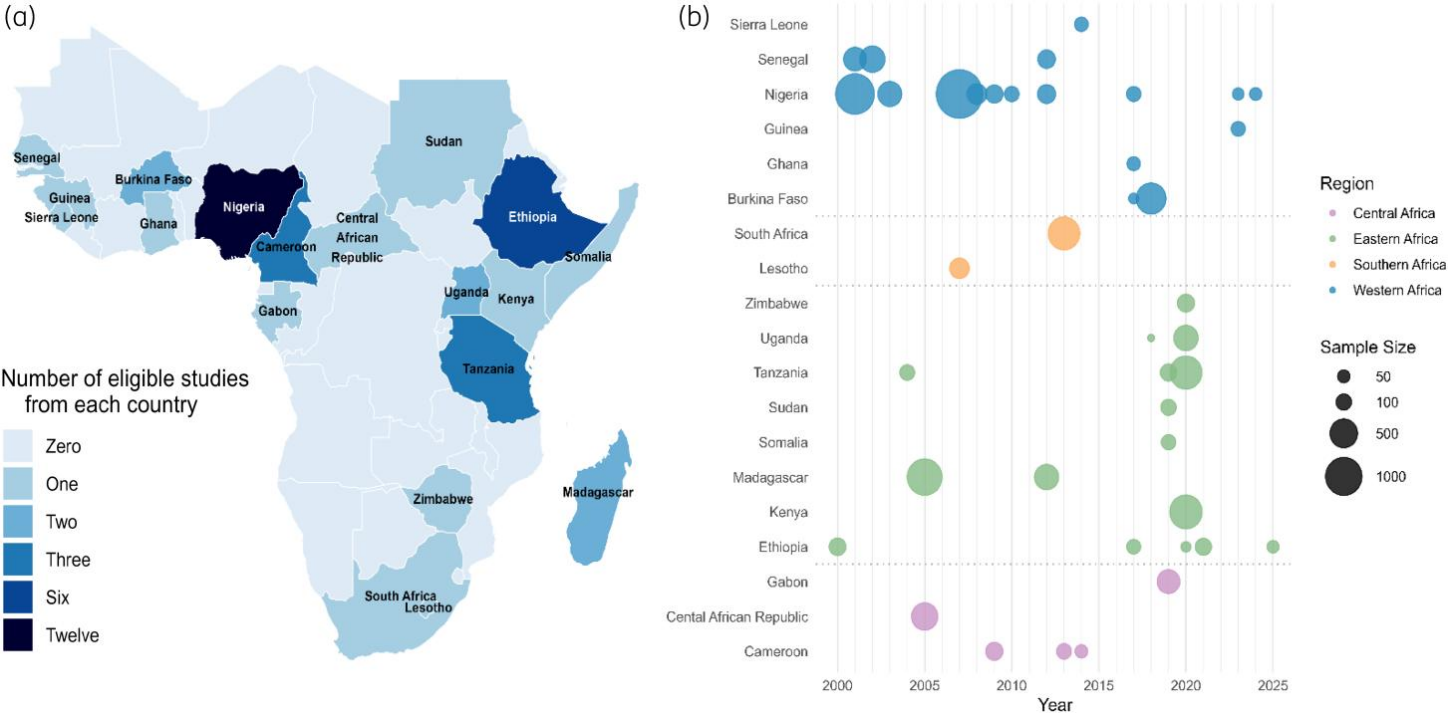
(a) Map showing eligible studies across countries in Sub-Saharan Africa. The legend scale indicates study density, where increasing shade intensitiy corresponds to a higher number of included studies per country (ranging from 0 to 12). (b) Bubble plot showing the geographical and temporal distribution of the included studies from 2000 to 2026. Studies are categorised by region (Western, Southern, Eastern and Central Africa along the y-axis) and study midpoint (x-axis). Circle size corresponds to total sample size of bacterial isolates from UTI patients.

Only 19 countries in Sub-Saharan Africa have published studies on CA-UTIs eligible for inclusion in this review (Figure 2a). Within this subset, research output was unevenly distributed, with the highest number of eligible studies originating from Nigeria (*n* = 12) and Ethiopia (*n* = 6). When stratified by region, Eastern Africa had the most nations represented with most of the research being conducted after 2015 (Figure 2b). Southern and Central Africa are least represented in this analysis.

Table 1 shows a summary of key study characteristics of all 42 included studies. Most of the included reports were prospective studies (83.3%) and disc diffusion was the most used AST methodology (92.9%). A broad range of UTI culture definitions were used as the cut-off for UTI positivity, showing a disagreement among laboratories; the most common UTI definition was 10^5^ colony forming units per millilitre (CFU/ml; 52.4% of included studies), however, 23.8% of all included studies did not provide any definition for UTI positivity. Two studies varied their UTI definition on the basis of which pathogen was responsible for the infection, and one mentioned a lower cut-off for young women compared with other patient populations.

**Table 1.** Study characteristics used for quality assessment

Quality variables		Number of studies (%)
Study design	Prospective	35 (83.3)
	Retrospective	6 (14.3)
	Case-control	1 (2.4)
Sample size	10–49	8 (19.0)
	50–99	14 (33.3)
	100–499	14 (33.3)
	500–999	3 (7.1)
	1000+	3 (7.1)
Antimicrobial Susceptibility method	disc diffusion	39 (92.9)
	disc diffusion + VITEK	1 (2.4)
	VITEK	1 (2.4)
	Other	1 (2.4)
UTI definition	≤10^3^ CFU/mL	1 (2.4)
	≥10^3^ CFU/mL	2 (4.8)
	≥10^4^ CFU/mL	4 (9.5)
	≥10^5^ CFU/mL	22 (52.4)
	Pathogen dependent	2 (4.8)
	Patient group dependent	1 (2.4)
	Not stated	10 (23.8)
AST guidelines	CLSI/NCCLS	23 (54.8)
	EUCAST	4 (9.5)
	CA-SFM	7 (16.7)
	Other	1 (2.4)
	Not stated	7 (16.7)
Internal quality control	Yes	22 (52.4)
	No	20 (47.6)

Thirty-five studies (83.3%) reported using formal AST guidelines in the interpretation of their results, while just over half (52.4%) of all studies reported the use of internal quality controls (Table 1): a practice considered essential by the International Organisation for Standardisation (ISO) 15189 laboratory standard.^27^ Furthermore, Clinical and Laboratory Standards Institute (CLSI) guidelines updated their breakpoints for certain antibiotics in 2011, results using the pre- and post-2011 criteria are reported separately.^28^

### *Meta-analysis of* E. coli *and* Klebsiella *spp. resistance*

*Klebsiella* spp. had higher resistance compared with *E. coli* for all antibiotics analysed (Table 2), except amoxicillin-clavulanic acid; however, this was not statistically significant (Table S4). The resistance to trimethoprim-sulfamethoxazole was significantly higher than nitrofurantoin, ceftriaxone or ciprofloxacin in *E. coli* strains (adjusted *P* < 0.006; Table S5); in addition, amoxicillin-clavulanic acid resistance was significantly higher than resistance to nitrofurantoin (adjusted *P* = 3.076 × 10^−4^). For *Klebsiella* spp., the only statistically significant difference in resistance was observed between nitrofurantoin [28.3%, 95% confidence interval (CI): 12.1%–44.5%] and trimethoprim-sulfamethoxazole (66.4%, 95%CI: 46.7%–86.2%; adjusted *P* = 0.034). Consistently across both pathogens, trimethoprim-sulfamethoxazole demonstrated the highest pooled resistance proportions (*E. coli:* 62.2%, 95%CI: 51.9%–72.5%; *Klebsiella* spp. 66.4%, 95%CI: 46.7%–86.2%), while nitrofurantoin maintained the lowest (*E. coli:* 20.3%, 95%CI: 10.4%–30.2%; *Klebsiella* spp.: 28.3%, 95%CI: 12.1%–44.5%).

**Table 2.** Results of meta-analysis showing the resistance of *E. coli* and *Klebsiella* spp. to five key antibiotics for UTI treatment

Organism	Antibiotic	Number of studies (isolates)	Pooled resistance prevalence (%)	95% confidence interval	*I* ^2^ statistic (%)
*E. coli*	Trimethoprim-sulfamethoxazole	22 (4888)	62.2	51.9–72.5	99.27
*Klebsiella* spp.	Trimethoprim-sulfamethoxazole	9 (609)	66.4	46.7–86.2	98.73
*E. coli*	Nitrofurantoin	24 (2656)	20.3	10.4–30.2	99.31
*Klebsiella* spp.	Nitrofurantoin	9 (743)	28.3	12.1–44.5	97.46
*E. coli*	Ciprofloxacin	31 (5602)	32.8	25.1–40.4	98.76
*Klebsiella* spp.	Ciprofloxacin	14 (907)	35.8	20.3–51.4	98.65
*E. coli*	Amoxicillin-clavulanic acid	30 (5898)	48.9	39.8–58.0	98.53
*Klebsiella* spp.	Amoxicillin-clavulanic acid	12 (768)	47.5	31.8–63.2	97.68
*E. coli*	Ceftriaxone	22 (3134)	34.5	22.4–46.6	99.28
*Klebsiella* spp.	Ceftriaxone	10 (733)	44.6	28.6–60.7	96.62

The percentage resistance is shown alongside the 95% CI and the sample size used to inform that number. Heterogeneity between studies is shown using the *I*^2^ statistic.

There was very high spatial variation in the reported resistance to each antibiotic for both *E. coli* and *Klebsiella spp.* (Figures 3–5; Figures S1–S7) across all included studies from Sub-Saharan Africa (*I*^2^ > 96.62%). Consequently, all pooled resistance proportions were calculated using a random-effects model to account for between-study variance.

**Figure 3.**
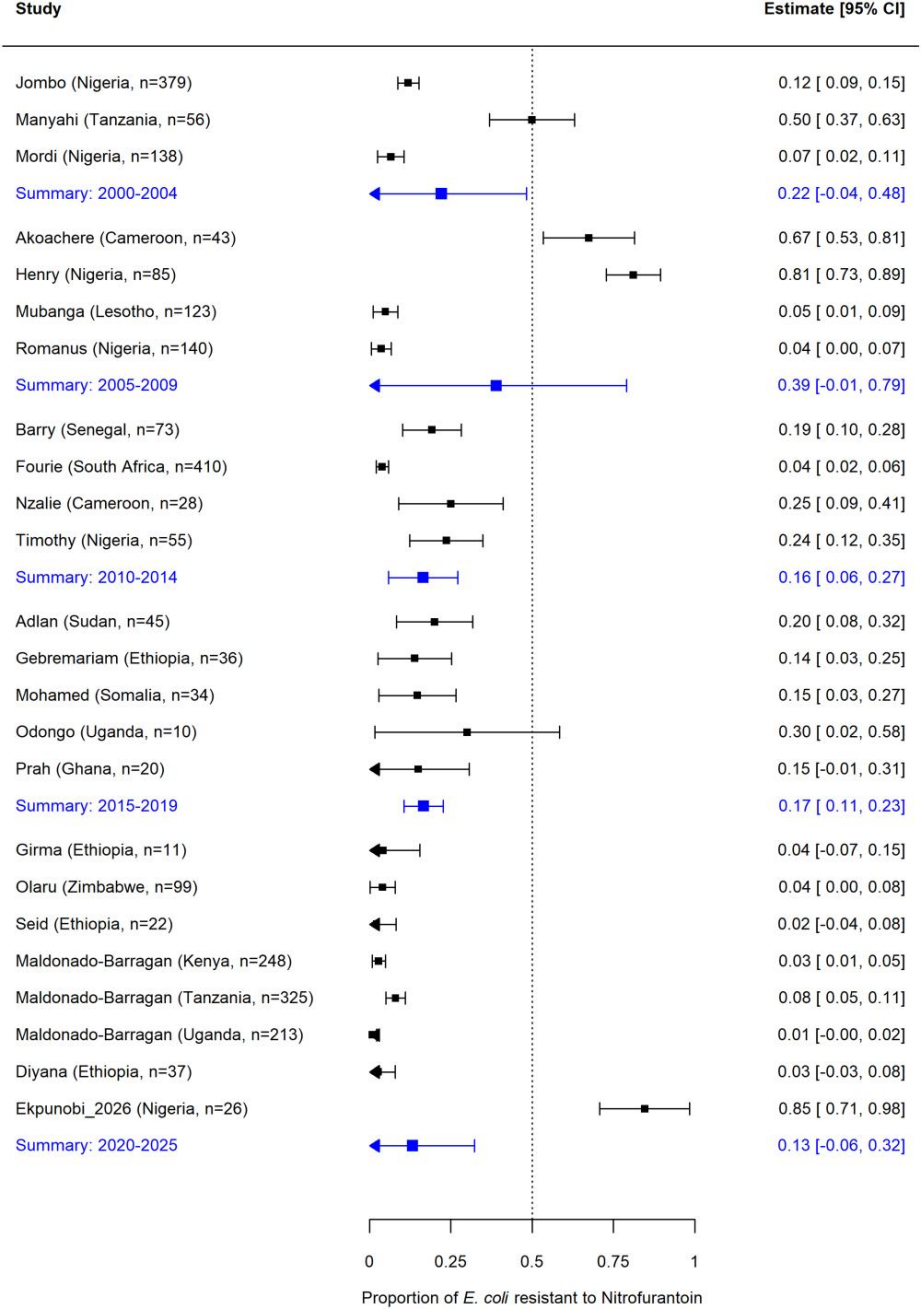
Forest plots illustrating the prevalence of nitrofurantoin-resistant *E. coli* strains from CA-UTI patients in Sub-Saharan Africa. Individual study results are presented alongside the country and sample size analysed (*n* =) per country with 95% CIs. The proportion of resistance is summarized (with 95% CIs) for each 5-year time block (e.g. 2000–2004, 2005–2009, 2010–2014, 2015–2019, 2020–2025) and this is shown in blue.

**Figure 4.**
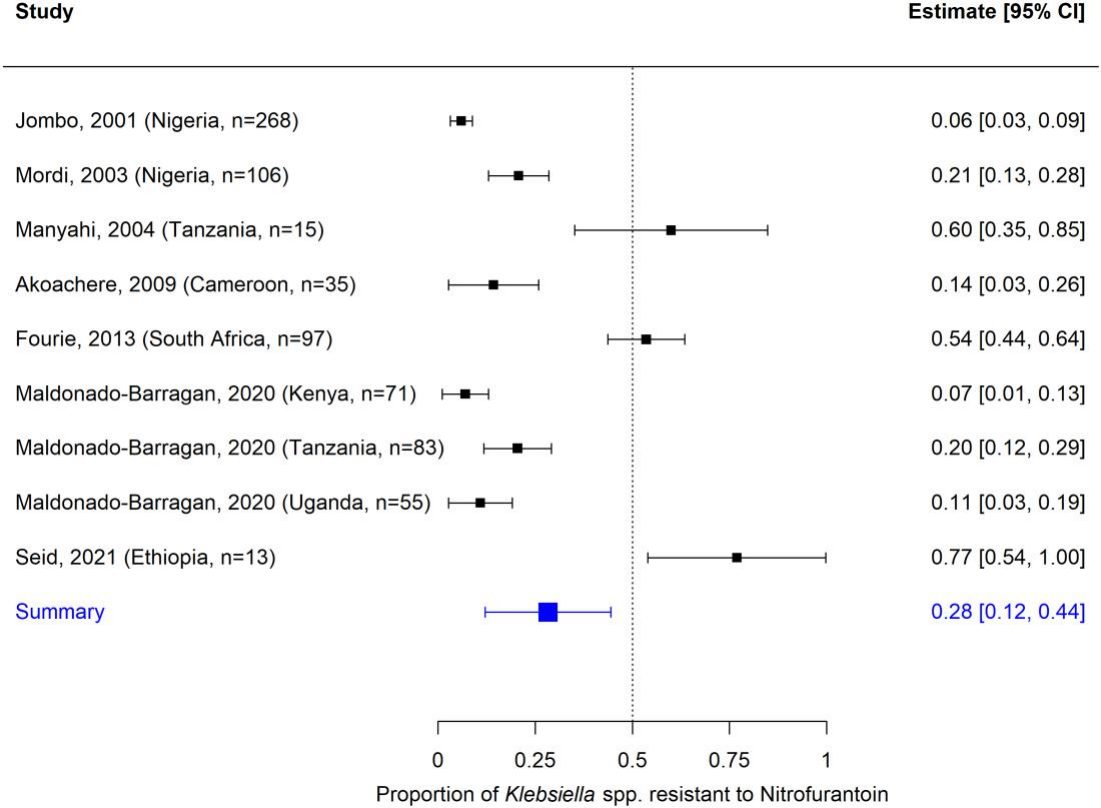
Forest plots illustrating the prevalence of nitrofurantoin resistance in *Klebsiella* spp. strains from CA-UTI patients in Sub-Saharan Africa. Individual study results are presented alongside the country and sample size analysed (*n* =) per country with 95% CI. The studies are ordered chronologically according to their study midpoint. The summary of nitrofurantoin resistance is shown in blue with 95% CIs.

**Figure 5.**
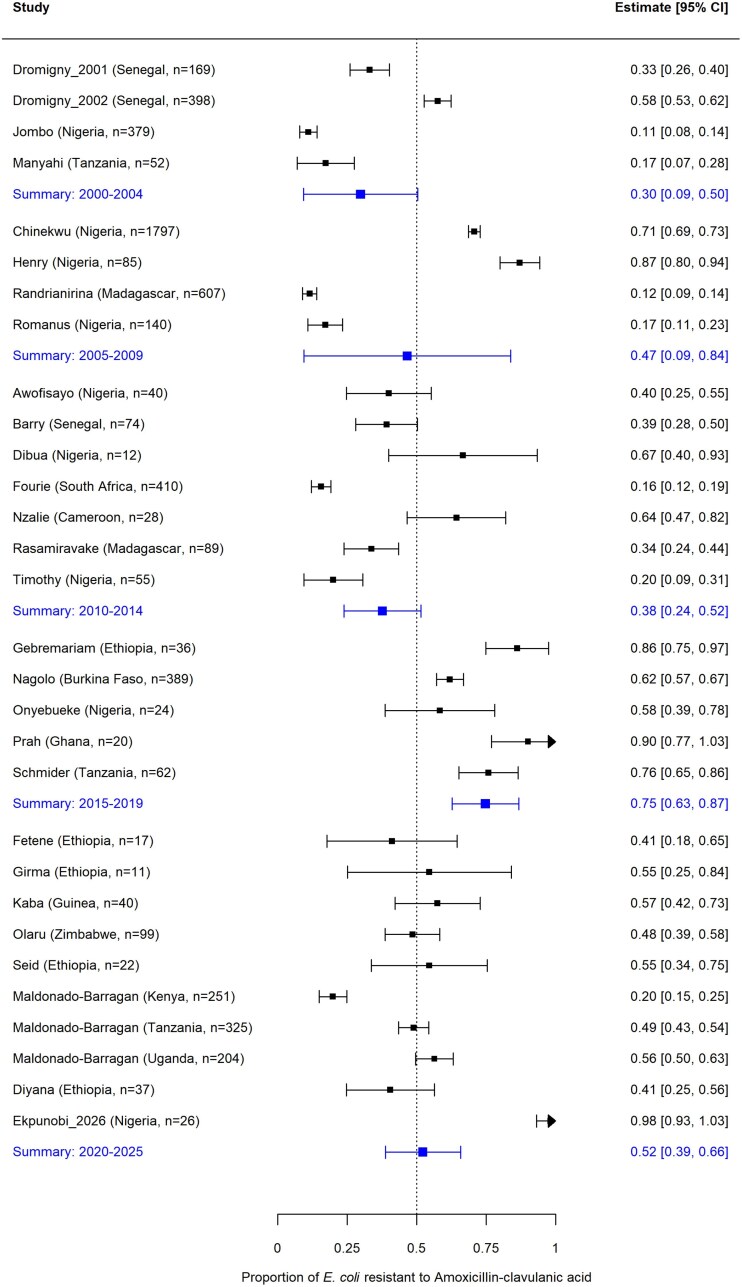
Forest plots illustrating the prevalence of amoxicillin-clavulanic acid resistant *E. coli* strains from CA-UTI patients in Sub-Saharan Africa. Individual study results are presented alongside the country and sample size analysed (*n* =) per country with 95% CIs. The proportion of resistance is summarized (with 95% CIs) for each 5-year time block (e.g. 2000–2004, 2005–2009, 2010–2014, 2015–2019, 2020–2025) and this is shown in blue.

*E. coli* forest plots showing the resistance rates to each antibiotic, were grouped into 5-year periods to explore whether resistance changed over the 25-year period (2000–2025) across Sub-Saharan Africa (Figures 3 and 5). However, this could not be analysed for *Klebsiella* spp. due to insufficient studies in each period (Figure 4). The forest plots highlight the heterogeneity of resistance prevalence for each organism-antibiotic pair.

There is a decrease in *E. coli* resistance to nitrofurantoin between 2000 and 2025 across Sub-Saharan Africa, however, meta-regression revealed that this was not a statistically significant decrease (*P* > 0.1346), potentially due to the high between-study variability. This heterogeneity is noticeable in *Klebsiella* spp. resistance to nitrofurantoin shown by the large 95% CIs in Figure 4 (12.1%–44.5%).

The only significant temporal shift in resistance was seen in 2015–2019 in *E. coli* resistance to amoxicillin-clavulanic acid (Figure 5); this was significantly higher than seen in the previous time block (*P* = 0.028). This trend did not continue into the 2020–2025-time block, as seen in the subgroup analysis (Table 3).

**Table 3.** Subgroup analysis for the pooled prevalence of *E. coli* resistance to ciprofloxacin, amoxicillin-clavulanic acid, trimethoprim-sulfamethoxazole, nitrofurantoin and ceftriaxone over time from 2000 to 2025; between western and eastern aAfrica; and between higher and lower quality studies. *Klebsiella* spp. data scarcity precluded providing results for all combinations. The pooled prevalence (with 95% CI) is reported alongside the *I*^2^ statistic, test statistic for the moderator (subgroup) effect (*Q*_M_ statistic) and the significance (shown using the *P* value, with significant results shown in bold)

Subgroup	Characteristics	Organism	Antibiotic	Number of studies	Number of isolates	Pooled prevalence (95% CI)	*I* ^2^ statistic	*Q*_M_ statistic	*P* value
Year group	2000–2004	*E. coli*	Ciprofloxacin	3	573	21.6 (−1.5–44.7)	97.181	3.703	0.448
	2005–2009			6	2795	21.6 (9.8–33.3)	97.864		
	2010–2014			8	720	35.3 (21.2–49.4)	92.884		
	2015–2019			7	575	39.0 (19.5–58.5)	95.078		
	2020–2025			7	939	39.1 (20.5–57.7)	97.221		
	2000–2004	*E. coli*	Amoxicillin-clavulanic acid	4	998	29.9 (9.4–50.4)	98.203	10.866	**0**.**028**
	2005–2009			4	2629	46.6 (9.4–83.7)	99.78		
	2010–2014			7	708	37.7 (23.9–51.6)	91.801		
	2015–2019			5	531	74.7 (62.7–86.7)	84.507		
	2020–2025			10	1032	52.3 (38.7–65.8)	95.791		
	2000–2004	*E. coli*	Trimethoprim-sulfamethoxazole	5	1140	67.9 (45.6–90.2)	99.273	1.401	0.844
	2005–2009			6	2795	58.0 (34.0–81.9)	99.471		
	2010–2014			5	652	53.9 (30.9–77.0)	96.939		
	2015–2019			3	143	65.4 (54.6–76.3)	47.415		
	2020–2025			3	158	72.8 (42.0–103.5)	95.114		
	2000–2004	*E. coli*	Nitrofurantoin	3	573	22.1 (−4.0–48.3)	98.774	2.835	0.586
	2005–2009			4	391	39.0 (−1.1–79.1)	99.577		
	2010–2014			4	566	16.5 (5.8–27.1)	84.244		
	2015–2019			5	145	16.6 (10.6–22.6)	0		
	2020–2025			8	981	13.2 (−5.9–32.3)	99.651		
	2010–2014	*E. coli*	Ceftriaxone	4	533	14.4 (5.8–23.1)	76.123	6.994	0.136
	2015–2019			5	524	48.7 (20.6–76.8)	98.122		
	2020–2025			9	992	43.7 (24.5–63.0)	98.344		
Region	Western Africa	*E. coli*	Ciprofloxacin	13	3165	32.0 (21.3–42.7)	98.593	3.362	0.339
	Eastern Africa			14	1833	38.2 (25.2–51.3)	97.366		
	Western Africa	*Klebsiella spp.*	Ciprofloxacin	5	485	39.6 (7.3–72.0)	99.4	1.017	0.797
	Eastern Africa			7	290	37.9 (16.8–59.0)	95.592		
	Western Africa	*E. coli*	Amoxicillin-clavulanic acid	15	3648	53.7 (39.8–67.6)	98.829	3.013	0.39
	Eastern Africa			13	1812	44.8 (32.5–57.2)	97.029		
	Western Africa	*Klebsiella spp.*	Amoxicillin-clavulanic acid	4	386	54.8 (16.4–93.2)	99.469	0.387	0.824
	Eastern Africa			7	285	43.4 (26.1–60.6)	90.501		
	Western Africa	*E. coli*	Trimethoprim-sulfamethoxazole	9	3231	62.9 (44.0–81.8)	99.72	3.005	0.391
	Eastern Africa			9	1053	69.7 (59.7–79.8)	90.97		
	Western Africa	*E. coli*	Nitrofurantoin	8	916	30.5 (7.8–53.2)	99.228	6.503	0.09
	Eastern Africa			12	1136	11.1 (3.6–18.6)	97.471		
	Western Africa	*E. coli*	Ceftriaxone	6	909	41.8 (14.5–69.1)	99.274	1.175	0.759
	Eastern Africa			13	1744	34.1 (18.6–49.5)	98.888		
Quality group	High quality (QS 1–2)	*E. coli*	Ceftriaxone	16	1816	39.7 (24.2–55.3)	99.077	1.927	0.165
	Low quality (QS 3–4)			6	1318	20.7 (9.0–32.4)	97.516		
	High quality (QS 1–2)	*E. coli*	Ciprofloxacin	21	4006	35.0 (25.4–44.6)	97.633	0.661	0.416
	Low quality (QS 3–4)			10	1596	28.1 (15.4–40.7)	98.348		
	High quality (QS 1–2)	*E. coli*	Amoxicillin-clavulanic acid	20	4302	51.1 (40.5–61.7)	98.277	0.417	0.518
	Low quality (QS 3–4)			10	1596	44.8 (27.0–62.5)	98.706		
	High quality (QS 1–2)	*E. coli*	Trimethoprim-sulfamethoxazole	15	3712	70.7 (62.1–79.3)	98.553	6.534	**0**.**011**
	Low quality (QS 3–4)			7	1176	45.4 (23.5–67.2)	98.978		
	High quality (QS 1–2)	*E. coli*	Nitrofurantoin	18	1542	19.0 (8.1–30.0)	99.102	0.158	0.691
	Low quality (QS 3–4)			6	1114	23.9 (0.5–47.2)	99.512		
	High quality (QS 1–2)	*Klebsiella*	Ceftriaxone	6	272	49.2 (31.6–66.8)	89.412	0.433	0.51
	Low quality (QS 3–4)			4	461	38.2 (6.4–70.1)	98.372		
	High quality (QS 1–2)	*Klebsiella*	Ciprofloxacin	9	428	36.1 (19.9–52.3)	93.985	0.002	0.964
	Low quality (QS 3–4)			5	479	35.4 (0.5–70.3)	99.588		
	High quality (QS 1–2)	*Klebsiella*	Amoxicillin-clavulanic acid	7	289	47.8 (35.9–59.8)	75.525	0.001	0.981
	Low quality (QS 3–4)			5	479	47.3 (12.6–82.0)	99.452		
	High quality (QS 1–2)	*Klebsiella*	Trimethoprim-sulfamethoxazole	6	226	84.3 (68.8–99.7)	96.851	19.02	**0**
	Low quality (QS 3–4)			3	383	33.3 (20.7–46.0)	76.829		

Publication bias analysis was examined using the Egger’s test, which showed that no significant bias in the resistance prevalence estimates except for *E. coli* resistance to nitrofurantoin (*P* = 0.0068), therefore a trim-and-fill sensitivity analysis was conducted for this organism-antibiotic combination (Table S6 and Figure S8). However, no missing studies were identified, and the pooled resistance estimate remained unchanged. This suggests that some small-study effects are present, but this does not significantly inflate the overall estimates. Multi-drug resistance (MDR) of *E. coli* and *Klebsiella* spp. could not be analysed as a variable as only three studies explicitly reported this.

### Subgroup analysis

Owing to the very high heterogeneity across the included studies for both *E. coli* and *Klebsiella* spp. antibiotic resistance prevalence, subgroup analysis was conducted. This analysis was used to investigate how the resistance prevalence changed spatially and temporally and whether study quality had any effect (Table 3). Subgroup analysis over time was not possible for *Klebsiella* spp. as there were too few studies to report this with sufficient granularity. High heterogeneity persisted in CA-UTI *E. coli* resistance when stratified by time (*I*^2^ > 75%), except for nitrofurantoin between 2015 and 2019 (*I*^2^ = 0) and trimethoprim-sulfamethoxazole resistance between 2015 and 2019 (*I*^2^ = 47.415). However, for both nitrofurantoin and trimethoprim-sulfamethoxazole in the 2015–2019 period, very few *E. coli* isolates were available to analyse (145 and 143, respectively).

This analysis showed that there was no significant difference in resistance rates over the 25 years (2000–2025) across Sub-Saharan Africa except for *E. coli* resistance to amoxicillin-clavulanic acid, which had a distinct peak in 2015–2019 (*Q*_M_ statistic = 10.866, *P* = 0.028). Owing to the limited dataset, only western and eastern Africa could be stratified in the subgroup analysis. Between these two regions, there was no significant difference in CA-UTI resistance (*E. coli P* ≥ 0.09; *Klebsiella* spp. *P* ≥ 0.797); suggesting that geographical region does not explain the large heterogeneity between studies.

To investigate whether laboratory methodological standardization affected the pooled resistance prevalence for either of the organisms, subgroup analysis was conducted between higher quality (quality score 1–2) and lower quality (quality score 3–4) studies (Table 3). The subgroup analysis based on quality score revealed that the reported resistance of *E. coli* and *Klebsiella* spp. to trimethoprim-sulfamethoxazole was significantly higher in studies that reported higher quality laboratory practices (Table 3; *E. coli: Q*_M_ = 6.534, *P* = 0.011; *Klebsiella* spp.: *Q*_M_ = 19.02, *P* = 0). However, for all other organism-antibiotic combinations the reported resistances did not differ significantly between the quality groups. IN addition, the heterogeneity within the higher and lower quality studies subgroups remained high (*I*^2^ > 75%). Last, analysis investigating the effect of the CLSI breakpoint changes in 2011, when the ceftriaxone disc-diffusion zone sizes were increased to lower the threshold for resistance,^28^ revealed that there was no significant difference in ceftriaxone resistance prevalence before or after this change (Table S7).

## Discussion

Synthesizing data from 42 studies across 19 countries over a 25-year period, this systematic review provides a comprehensive examination of phenotypic resistance in acute CA-UTIs in Sub-Saharan Africa. By evaluating 6308 *E. coli* and 930 *Klebsiella* spp. isolates collected, we examined the resistance patterns for five frontline antibiotics: ciprofloxacin, nitrofurantoin, amoxicillin-clavulanic acid, trimethoprim-sulfamethoxazole and ceftriaxone. Given that these pathogens cause >80% of all uncomplicated UTIs,^7^ characterizing their susceptibility profiles is critical for informing empirical treatment guidelines. This systematic analysis demonstrates the extensive prevalence of AMR in CA-UTI patients across Sub-Saharan Africa.

Despite most acute UTI cases originating and being treated in the community,^29,30^ there is very little information on the global AMR burden in this patient population.^31^ In Sub-Saharan Africa, where antimicrobial consumption is less regulated than in higher income regions and several countries have outdated STGs, it is vital to understand the burden of resistance to treat patients effectively and reduce the transmission of AMR.^10,32^

This review reveals a distinct lack of concrete data on the prevalence of resistance in bacteria causing CA-UTI in patients across Sub-Saharan Africa, with most of this region having no eligible studies for inclusion (Figure 2a). There was also significant variation in the resistance prevalence reported between the included studies (*I*^2^ > 96%), which is reflected in the large CIs (Table 2) for each pooled resistance estimate. This limits this review’s ability to accurately estimate the AMR rates of bacteria causing CA-UTIs across this region. This high heterogeneity is also seen in other reviews and could not be resolved by geographical or temporal subgrouping (Table 3).^33^

Overall, nitrofurantoin resistance rates were the lowest of all antibiotics tested both for *E. coli* and *Klebsiella* spp. at 20.3% (95%CI: 10.4%–30.2%) and 28.3% (95% CI: 12.1%–44.5%), respectively (Figures 3 and 4). This is higher than other published estimates that have reported a pooled nitrofurantoin resistance prevalence of 6.9% globally and 9.3% in Africa for uropathogenic *E. coli.*^34^ However, this review did not specifically select for community-acquired UTI patients and only included 16 African studies, which may explain the difference in resistance estimates. Furthermore, temporal subgroup analysis indicated a decline in the prevalence of nitrofurantoin-resistant *E. coli* in more recent study periods (from 22.1% to 13.2%; Table 3). Although this reduction did not reach statistical significance, the absence of an upward resistance trajectory highlights the continued clinical utility of this antimicrobial agent, which echoes what is seen in other countries and regions.

The pooled prevalence of ciprofloxacin-resistant *E. coli* and *Klebsiella* spp. was high at 32.8% (95%CI: 25.1%–40.4%) and 35.8% (95%CI: 20.3%–51.4%) in bacteria isolated from CA-UTI patients (Figures S2 and S6). These findings are similar to other reports in the literature that state a prevalence of ciprofloxacin resistance of 34% in African UTI patients or 36.1% for CA-UTI patients in Africa, Asia and the Middle East.^33,35^ Fasugba *et al*.’s global review found a similarly large variation in the resistance reported within bacteria isolated from the community (*I*^2^ = 98.8%) and saw an increase in the resistance over time.^33^ Subgroup analysis shows an increase in ciprofloxacin resistance across the 5-year groupings; however, this was not statistically significant (Table 3). Therefore, local ciprofloxacin resistance rates and the propensity for ciprofloxacin resistance to be transmitted easily should be considered before using this alternative treatment.^10^

The prevalence of *E. coli* resistance to amoxicillin-clavulanic acid in this review is on the lower end of Bryce *et al*.’s estimated resistance in non-member countries of the Organisation for Economic Cooperation and Development (OECD) which reported 60.8% (95% CI: 40.9%–79%) resistance in paediatric UTI patients.^36^ The pooled resistance prevalence of 48.9% (95% CI: 39.8%–58.0%) for *E. coli* and 47.5% (95% CI: 31.8%–63.2%) for *Klebsiella spp.*, is very similar between both species (Figures 5 and S4). However, this high resistance rate is concerning for its recommendation as a first-line treatment for acute UTIs, as there is a high chance of clinical failure.^10,11^

Ceftriaxone resistant isolates frequently exhibit a MDR phenotype, typically driven by the acquisition of plasmids containing extended-spectrum β-lactamase genes alongside other resistance genes, which severely limits subsequent treatment options.^37^ While ceftriaxone is primarily indicated in complicated UTI or pyelonephritis treatment, monitoring its resistance in the community is critical given its essential role as a second-line treatment.^11^ Ceftriaxone resistance was higher in *Klebsiella* spp. at 44.6% (95% CI: 28.6%–60.7%) than *E. coli* at 34.5% (95% CI: 22.4%–46.6%; Figures S3 and S7), which corroborates findings in bloodstream isolates from Sub-Saharan Africa.^38^ This alarmingly high rate of resistance is probably driven by the fact that ceftriaxone is inappropriately prescribed to 60.8% of patients in this global region.^39^ Ceftriaxone resistance breakpoints shifted drastically in the CLSI M100 2011 revisions, however, this did not have a significant effect on the resistance prevalence (Table S7), showing that the high resistance prevalence is not an artefact of shifting diagnostic cut-offs.^28^

The antibiotic with the highest pooled resistance prevalence was trimethoprim-sulfamethoxazole with 62.2% (95% CI: 51.9%–72.5%) and 66.4% (95% CI: 46.7%–86.2%) for *E. coli* and *Klebsiella* spp., respectively (Figures S1 and S5). Bryce *et al.* reported a similarly high resistance in paediatric UTIs in non-OECD countries at 69.6%, however, in OECD countries the resistance was lower at 30.2%.^36^ Trimethoprim-sulfamethoxazole prophylaxis is recommended for individuals with HIV, which may affect the resistance rates seen to this treatment within the community.^40,41^ This supports the removal of this treatment from the UTI African Antibiotic Treatment guidelines, as resistance rates are too high for it to be efficacious without knowing the local resistance rates.^10,14^

Subgroup analysis showed a non-significant difference in the prevalence of *E. coli* resistance between eastern and western Africa, which is in line with UTI ciprofloxacin resistance mapped by Kibwana *et al.*^35^ A review on AMR across Africa found that western and eastern Africa had the highest total AMR prevalence out of the four African regions (at 40.5% and 42.5%, respectively), with southern Africa having the lowest (at 33.9%), if more data from southern Africa had existed, this review may have seen a regional shift.^42^ CA-UTI resistance prevalence did not change significantly between 2000 and 2025 except for a spike in amoxicillin-clavulanic acid resistance between 2015 and 2019 that then decreased in the subsequent time block (Table 3). A potential explanation for this increase is the pivot away from empirical fluoroquinolone use following clinical guideline amendments, which increased the use of amoxicillin-clavulanic acid and thus the selective pressure on resistance development.^11,43^

The only significant difference between higher and lower quality papers was the prevalence of *E. coli* and *Klebsiella* spp. resistance to trimethoprim-sulfamethoxazole (Table 3). This is most likely because the lower quality subgroup is dominated by two large retrospective studies (accounting for 67.1% of total isolates in the subgroup) that did not have granular information of UTI culture cut-offs or AST guidelines.^44,45^

Despite not being the primary aim of this review, one of the most notable conclusions is the distinct lack of standardization in UTI laboratory reporting (Table 1). The variety of UTI culture cut-offs used in the included studies is not unique to Sub-Saharan African studies but reflects a wider lack of consensus in UTI research.^46^ This variation, alongside a lack of AST guideline and internal quality control reporting, may have introduced bias into the pooled AMR prevalences in this review. Therefore, in addition to needing more data across Sub-Saharan Africa on the AMR prevalence of CA-UTIs there is an urgent need for standardization in UTI reporting so that AMR rates can be accurately compared globally.

### Limitations

The main limitation of the systematic review was a lack of eligible studies in 30 Sub-Saharan African countries and the small number of positive samples described in the studies. This limits the strength of any conclusions about resistance rates of CA-UTIs across this region and highlights the major gap in our understanding. It is impossible to infer country-level CA-UTI AMR prevalence from one small study in one area of a country. A significant challenge in this meta-analysis was the limited sample size of 42 studies, which restricted our ability to extensively control for compounding variables. Conducting multi-variable subgroup analyses to isolate geographic, temporal and methodological differences would have fractured the dataset into subgroups of one or two studies, rendering further statistical testing unreliable.

Furthermore, a notable limitation was the inability to stratify AMR rates by key demographic factors, such as age or sex. Analysing the population differences in AMR between paediatric and adult CA-UTI isolates, or between male and female populations, would have enriched the outputs of this review. However, disaggregated AST results were not available from any of the primary studies, meaning that demographically stratified subgroup meta-analysis was unfeasible.

Included studies only listed the number of sensitive, intermediate or resistant isolates to specific antibiotics, therefore we were unable pull the data together to quantify the proportion of MDR within CA-UTI patients in Sub-Saharan Africa. Furthermore, the disc-diffusion diameters were not provided so it was not possible to standardize the AST designation which differs between guidelines over time.

This review focuses only on acute UTIs within the community and excludes studies with complicated UTI patients or those with comorbidities. This was necessary as patients with comorbidities suffer from complicated UTIs and may undergo different treatment regimens.^47^ Subsequently, including these patients may have biased the analysis as certain patient populations may have an increased risk of developing AMR. However, it also meant that papers that reported fewer patient variables (who excluded information on pregnancy or additional health data on patients) and fewer methodology controls or guidelines may well have inadvertently been included due to lack of information. Many studies recruited their CA-UTI patients from outpatient departments or new hospital admissions (<48–72 h) that resulted in less representation of CA-UTIs from primary clinics or pharmacies (Table S3). Outpatients with UTI symptoms were used as a proxy for CA-UTI as this was the only information available, however, this may have inadvertently captured some patients with recent healthcare exposure.

In conclusion, the resistance in CA-UTIs varies dramatically between studies across Sub-Saharan Africa highlighting the need to understand local resistance rates within the community to inform prescribing practices and antimicrobial stewardship. International clinical practice guidelines advise empirical treatment if the local resistance rate of uropathogens is <20%, therefore data from this review highlight the urgency for coordinated antimicrobial stewardship efforts alongside regular AMR surveillance in the community.^14,48^ Novel point-of-care tests that can inform antibiotic treatments quickly in community settings are vital to ensure these treatments can continue to be used for UTIs where the antibiotics will be effective.

## Supplementary Material

dlag155_Supplementary_Data

## Supplementary data

Figures S1 to S8 and Tables S1 to S7 are available as Supplementary data at *JAC-AMR* Online.
