# Supplementary material for: Prevalence of antimicrobial resistance in acute community-acquired urinary tract infections in Sub-Saharan Africa: a systematic review
Source: JAC Antimicrob Resist. 2026 Aug 17;8(4):dlag155. doi: 10.1093/jacamr/dlag155 (PMC13479187; doi:10.1093/jacamr/dlag155)
Supplement: Supplementary file 1 [file dlag155_supplementary_data.docx]

**PRISMA 2020 Checklist**

| **Section and Topic** | **Item #** | **Checklist item** | **Location where item is reported** |
| --- | --- | --- | --- |
| **TITLE** | | |  |
| Title | 1 | Identify the report as a systematic review. | Title |
| **ABSTRACT** | | |  |
| Abstract | 2 | See the PRISMA 2020 for Abstracts checklist. | Abstract |
| **INTRODUCTION** | | |  |
| Rationale | 3 | Describe the rationale for the review in the context of existing knowledge. | Introduction paragraph 3-4 |
| Objectives | 4 | Provide an explicit statement of the objective(s) or question(s) the review addresses. | Introduction paragraph 5 |
| **METHODS** | | |  |
| Eligibility criteria | 5 | Specify the inclusion and exclusion criteria for the review and how studies were grouped for the syntheses. | Methods subsection “Selection criteria” |
| Information sources | 6 | Specify all databases, registers, websites, organisations, reference lists and other sources searched or consulted to identify studies. Specify the date when each source was last searched or consulted. | Methods subsection “Search Strategy” |
| Search strategy | 7 | Present the full search strategies for all databases, registers and websites, including any filters and limits used. | Supplementary material 2 |
| Selection process | 8 | Specify the methods used to decide whether a study met the inclusion criteria of the review, including how many reviewers screened each record and each report retrieved, whether they worked independently, and if applicable, details of automation tools used in the process. | Methods subsection “Search strategy” |
| Data collection process | 9 | Specify the methods used to collect data from reports, including how many reviewers collected data from each report, whether they worked independently, any processes for obtaining or confirming data from study investigators, and if applicable, details of automation tools used in the process. | Methods subsection “Data extraction” |
| Data items | 10a | List and define all outcomes for which data were sought. Specify whether all results that were compatible with each outcome domain in each study were sought (e.g. for all measures, time points, analyses), and if not, the methods used to decide which results to collect. | Methods subsection “Data extraction” |
|  | 10b | List and define all other variables for which data were sought (e.g. participant and intervention characteristics, funding sources). Describe any assumptions made about any missing or unclear information. | Methods subsection “Data extraction” |
| Study risk of bias assessment | 11 | Specify the methods used to assess risk of bias in the included studies, including details of the tool(s) used, how many reviewers assessed each study and whether they worked independently, and if applicable, details of automation tools used in the process. | Methods subsection “Quality assessment” |
| Effect measures | 12 | Specify for each outcome the effect measure(s) (e.g. risk ratio, mean difference) used in the synthesis or presentation of results. | Methods subsection “Data analysis” |
| Synthesis methods | 13a | Describe the processes used to decide which studies were eligible for each synthesis (e.g. tabulating the study intervention characteristics and comparing against the planned groups for each synthesis (item #5)). | Methods subsection “Data analysis” |
|  | 13b | Describe any methods required to prepare the data for presentation or synthesis, such as handling of missing summary statistics, or data conversions. | Methods subsection “Data analysis” |
|  | 13c | Describe any methods used to tabulate or visually display results of individual studies and syntheses. | Methods subsection “Data analysis” |
|  | 13d | Describe any methods used to synthesize results and provide a rationale for the choice(s). If meta-analysis was performed, describe the model(s), method(s) to identify the presence and extent of statistical heterogeneity, and software package(s) used. | Methods subsection “Data analysis” |
|  | 13e | Describe any methods used to explore possible causes of heterogeneity among study results (e.g. subgroup analysis, meta-regression). | Methods subsection “Data analysis” |
|  | 13f | Describe any sensitivity analyses conducted to assess robustness of the synthesized results. | N/A |
| Reporting bias assessment | 14 | Describe any methods used to assess risk of bias due to missing results in a synthesis (arising from reporting biases). | Methods subsection “Data analysis” |
| Certainty assessment | 15 | Describe any methods used to assess certainty (or confidence) in the body of evidence for an outcome. | Methods subsection “Data analysis” |
| **RESULTS** | | |  |
| Study selection | 16a | Describe the results of the search and selection process, from the number of records identified in the search to the number of studies included in the review, ideally using a flow diagram. | Figure 1 |
|  | 16b | Cite studies that might appear to meet the inclusion criteria, but which were excluded, and explain why they were excluded. | Figure 1 (and associated text) |
| Study characteristics | 17 | Cite each included study and present its characteristics. | Supplementary Information S3; Table 1 |
| Risk of bias in studies | 18 | Present assessments of risk of bias for each included study. | Supplementary Information S2/S3 |
| Results of individual studies | 19 | For all outcomes, present, for each study: (a) summary statistics for each group (where appropriate) and (b) an effect estimate and its precision (e.g. confidence/credible interval), ideally using structured tables or plots. | Forest plots (Figure 3-5 and S7-13) |
| Results of syntheses | 20a | For each synthesis, briefly summarise the characteristics and risk of bias among contributing studies. | Table 1 |
|  | 20b | Present results of all statistical syntheses conducted. If meta-analysis was done, present for each the summary estimate and its precision (e.g. confidence/credible interval) and measures of statistical heterogeneity. If comparing groups, describe the direction of the effect. | Table 2 |
|  | 20c | Present results of all investigations of possible causes of heterogeneity among study results. | Subgroup analysis: Table 3 |
|  | 20d | Present results of all sensitivity analyses conducted to assess the robustness of the synthesized results. | N/A |
| Reporting biases | 21 | Present assessments of risk of bias due to missing results (arising from reporting biases) for each synthesis assessed. | Supplementary Information S13 |
| Certainty of evidence | 22 | Present assessments of certainty (or confidence) in the body of evidence for each outcome assessed. | Supplementary Information S13 |
| **DISCUSSION** | | |  |
| Discussion | 23a | Provide a general interpretation of the results in the context of other evidence. | Discussion paragraphs 4-9 |
|  | 23b | Discuss any limitations of the evidence included in the review. | Limitations |
|  | 23c | Discuss any limitations of the review processes used. | Limitations |
|  | 23d | Discuss implications of the results for practice, policy, and future research. | Discussion paragraphs 10 and 14 |
| **OTHER INFORMATION** | | |  |
| Registration and protocol | 24a | Provide registration information for the review, including register name and registration number, or state that the review was not registered. | Methods paragraph 1 |
|  | 24b | Indicate where the review protocol can be accessed, or state that a protocol was not prepared. | Methods paragraph 1 |
|  | 24c | Describe and explain any amendments to information provided at registration or in the protocol. | Methods paragraph 1 |
| Support | 25 | Describe sources of financial or non-financial support for the review, and the role of the funders or sponsors in the review. | N/A |
| Competing interests | 26 | Declare any competing interests of review authors. | N/A |
| Availability of data, code and other materials | 27 | Report which of the following are publicly available and where they can be found: template data collection forms; data extracted from included studies; data used for all analyses; analytic code; any other materials used in the review. | Supplemental Information |

*From:* Page MJ, McKenzie JE, Bossuyt PM, Boutron I, Hoffmann TC, Mulrow CD, et al. The PRISMA 2020 statement: an updated guideline for reporting systematic reviews. BMJ 2021;372:n71. doi: 10.1136/bmj.n71

Table S1: The search strategy used for Embase, Medline and Global Health databases

Embase <1996 to 2026 Week 17>

Ovid MEDLINE(R) <1996 to April Week 3 2026>

Global Health <1973 to 2026 Week 17>

| **Search terms** | **Number of studies** |
| --- | --- |
| 1 ("Urinary tract infectio*" or "urine infection*" or UTI or UTIs or cystitis or pyelonephri* or urethriti* or uropathogen* or "bladder infectio*" or "uncomplicated urinary tract infection" or "acute urinary tract infection" or bacteriuria* or pyuria*).ti,ab. | 195939 |
| 2 ("community-onset" or "community-aquir*" or communit* or outpatien* or "primary care" or household or "out-patien*" or clinic* or "ambulatory care" or "community-associate*" or "community-base*" or ambulatory or "primary-care" or "primary health").mp | 23099952 |
| 3 ("Sub-Saharan Africa" or "Southern Africa" or "Africa south of the sahara" or "Africa south of sahara" or "Low middle income countr*" or LMICs or SSA or Angola or Benin or Botswana or "Burkina Faso" or Burundi or "Cabo Verde" or "Cape Verde" or Cameroon or "Central African Republic" or "Central African Empire" or Chad or Tchad or Comoros or Comores or Congo or DRC or "Cote d'Ivoire" or "Ivory Coast" or "Equatorial Guinea" or Eritrea or Eswatini or Swaziland or Ethiopia or Gabon or "Gabonese Republic" or Gambia or Ghana or Guinea or "Guinea-Bissau" or Kenya or Lesotho or Liberia or Madagascar or Malawi or Mali or Mauritani* or Mauritius or Mozambique or Namibia or Niger or Nigeria or Rwanda or "Sao Tome and Principe" or Senegal or Seychell* or "Sierra Leone" or Salone or Somali* or "South Africa" or "South Sudan" or Sudan or Tanzania or Zanzibar or Togo or Togol* or Uganda or Zambia or Zimbabwe or Rhodesia).ti,ab | 1207555 |
| 1 and 2 and 3 | 3099 |

**Table S2: Risk of Bias reporting, an adaptive methodological assessment of study quality**

| **Quality Grade** | **Methodological Criteria** | **Data Integrity Requirements** |
| --- | --- | --- |
| **Group 1 (Highest)** | Explicit UTI definition AND adherence to AST guidelines AND documented internal Quality Control (QC). | No missing data and consistent reporting |
| **Group 2** | Fulfilment of any two of the following: UTI definition, AST guidelines, or internal QC. | No missing data and consistent reporting |
| **Group 3** | Fulfilment of only one of the following: UTI definition, AST guidelines, or internal QC. | No missing data and consistent reporting |
| **Group 4 (Lowest)** | Absence of UTI definition, AST guidelines, AND internal QC. | Presence of missing or inconsistent data. |

**Table S3: Summary of study characteristics including the author, year of publication, country of study, location of recruitment, number of isolates and quality score**

| **Author** | **Year** | **Country** | **Location of recruitment** | **Number of isolates** | **Quality score** |
| --- | --- | --- | --- | --- | --- |
| Barry | 2017 | Senegal | Outpatient/Inpatient department of hospitals if admitted for less than 72hrs and Private primary care clinics in Dakar | 147 | 1 |
| Bercion | 2009 | Cental African Republic | Outpatient department from Institut Pasteru de Bangui | 443 | 1 |
| Chinekwu | 2013 | Nigeria | Outpatient department from referral public hospital and private clinic | 1797 | 1 |
| Diyana | 2026 | Ethiopia | Outpatient department of referral hospital | 47 | 1 |
| Dromigny | 2003 | Senegal | Outpatients from private lab, referal hospital and IPD | 300 | 1 |
| Dromigny | 2005 | Senegal | Outpatient department at Institut Pasteur de Dakar | 398 | 1 |
| Gebremariam | 2019 | Ethiopia | University clinic | 74 | 1 |
| Girma | 2022 | Ethiopia | Outpatient department of public referral hospital | 28 | 1 |
| Kiemde | 2020 | Burkina Faso | Outpatient department of large referral hospital | 29 | 1 |
| Maldonado-Barragan; Kiiru | 2024;2023 | Kenya, Tanzania, Uganda | Outpatients from hospitals and clinics | 1856 | 1 |
| Olaru | 2021 | Zimbabwe | 10 Primary healthcare clinics | 136 | 1 |
| Prah | 2019 | Ghana | Outpatient department of referral hospital | 64 | 1 |
| Randrianirina | 2007 | Madagascar | Outpatient department in Institut Pasteur Madigascar | 903 | 1 |
| Rasamiravake | 2015 | Madagascar | Retrospective study of general community samples | 357 | 1 |
| Seid | 2023 | Ethiopia | Outpatient department of referral hospital | 65 | 1 |
| Akoachere | 2012 | Cameroon | Outpatient/Inpatient department of hospitals if admitted less than 48hrs before | 137 | 2 |
| Ekpunobi | 2026 | Nigeria | Outpatient department of referral hospital | 49 | 2 |
| Fetene | 2024 | Ethiopia | Student clinic | 51 | 2 |
| Kaba | 2024 | Guinea | Outpatient department of large referral hospital | 75 | 2 |
| Leski | 2016 | Sierra Leone | Outpatient department of small private hospital | 70 | 2 |
| Lyonga | 2015 | Cameroon | Outpatient department from large referral hospital | 89 | 2 |
| Manyahi | 2017 | Tanzania | Outpatient department of large referral hospital | 88 | 2 |
| Mohamed | 2020 | Somalia | Outpatient department | 83 | 2 |
| Mordi | 2006 | Nigeria | Outpatient clinics of referral hospital | 347 | 2 |
| Mouanga-Ndzime | 2023 | Gabon | Child community samples sent to laboratory | 304 | 2 |
| Mubanga | 2015 | Lesotho | Outpatient department of referral hospital | 200 | 2 |
| Nzalie | 2016 | Cameroon | Outpatients and inpatients of less than 48hrs | 55 | 2 |
| Odongo | 2020 | Uganda | Outpatient department of large referral hospital | 17 | 2 |
| Otajevwo | 2013 | Nigeria | Outpatient department of large referral hospital | 36 | 2 |
| Adlan | 2021 | Sudan | Outpatient clinics of state hospitals | 99 | 3 |
| Ekpunobi | 2024 | Nigeria | Outpatients from diagnostic centre | 41 | 3 |
| Fourie | 2021 | South Africa | Outpatient, university community clinic and casualty | 712 | 3 |
| Henry | 2011 | Nigeria | Community patients in hospital | 204 | 3 |
| Onyebueke | 2020 | Nigeria | University clinic | 80 | 3 |
| Romanus | 2011 | Nigeria | Outpatient department of referral hospital | 140 | 3 |
| Schmider | 2022 | Tanzania | Outpatient department of referral hospital | 119 | 3 |
| Timothy | 2014 | Nigeria | Outpatient departments of referral hospital | 84 | 3 |
| Awofisayo | 2014 | Nigeria | Outpatient department of large referral hospital | 80 | 4 |
| Dibua | 2014 | Nigeria | Outpatient department | 43 | 4 |
| Jombo | 2006 | Nigeria | Outpatient department or those admitted for less than 24hrs to large referral hospital | 1169 | 4 |
| Moges | 2002 | Ethiopia | Outpatient department of large referral hospital | 127 | 4 |
| Nagolo | 2024 | Burkina Faso | Outpatients and inpatients of less than 48hrs | 614 | 4 |

**Table S4:** Z-test with Bonferroni correction comparing resistance prevalence between *E. coli* and *Klebsiella* spp.

| **Antibiotic** | ***E. coli* resistance** | ***Klebsiella* spp. resistance** | **Standard error (*E. coli)*** | **Standard error (*Klebsiella* spp.)** | **Z score** | **Raw p-value** | **Bonferroni adjusted p-value** |
| --- | --- | --- | --- | --- | --- | --- | --- |
| Trimethoprim-sulfamethoxazole | 0.622 | 0.664 | 0.052 | 0.101 | -0.370 | 0.712 | 1 |
| Nitrofurantoin | 0.203 | 0.283 | 0.05 | 0.082 | -0.833 | 0.405 | 1 |
| Ciprofloxacin | 0.328 | 0.358 | 0.039 | 0.079 | -0.341 | 0.733 | 1 |
| Amoxicillin-clavulanic acid | 0.489 | 0.475 | 0.047 | 0.08 | 0.151 | 0.880 | 1 |
| Ceftriaxone | 0.345 | 0.446 | 0.062 | 0.082 | -0.982 | 0.326 | 1 |

**Table S5:** Z-test with Bonferroni correction comparing the resistance between each antibiotic pair for each organism analysed.

| **Organism** | **Antibiotic 1** | **Antibiotic 2** | **Estimate 1** | **Estimate 2** | **Z score** | **Raw p-value** | **Bonferroni adjusted p-value** |
| --- | --- | --- | --- | --- | --- | --- | --- |
| *E. coli* | Trimethoprim-sulfamethoxazole | Nitrofurantoin | 0.622 | 0.203 | 5.808 | 6.313E-09 | 6.313E-08 |
| *E. coli* | Trimethoprim-sulfamethoxazole | Ceftriaxone | 0.622 | 0.345 | 3.423 | 0.001 | 0.006 |
| *E. coli* | Trimethoprim-sulfamethoxazole | Ciprofloxacin | 0.622 | 0.328 | 4.523 | 6.095E-06 | 6.095E-05 |
| *E. coli* | Trimethoprim-sulfamethoxazole | Amoxicillin-clavulanic acid | 0.622 | 0.489 | 1.897 | 0.058 | 0.578 |
| *E. coli* | Nitrofurantoin | Ceftriaxone | 0.203 | 0.345 | -1.783 | 0.075 | 0.746 |
| *E. coli* | Nitrofurantoin | Ciprofloxacin | 0.203 | 0.328 | -1.971 | 0.049 | 0.487 |
| *E. coli* | Nitrofurantoin | Amoxicillin-clavulanic acid | 0.203 | 0.489 | -4.168 | 3.076E-05 | 3.076E-04 |
| *E. coli* | Ceftriaxone | Ciprofloxacin | 0.345 | 0.328 | 0.232 | 0.816 | 1.000 |
| *E. coli* | Ceftriaxone | Amoxicillin-clavulanic acid | 0.345 | 0.489 | -1.851 | 0.064 | 0.642 |
| *E. coli* | Ciprofloxacin | Amoxicillin-clavulanic acid | 0.328 | 0.489 | -2.636 | 0.008 | 0.084 |
| *Klebsiella* spp. | Trimethoprim-sulfamethoxazole | Nitrofurantoin | 0.664 | 0.283 | 2.929 | 0.003 | 0.034 |
| *Klebsiella* spp. | Trimethoprim-sulfamethoxazole | Ceftriaxone | 0.664 | 0.446 | 1.676 | 0.094 | 0.938 |
| *Klebsiella* spp. | Trimethoprim-sulfamethoxazole | Ciprofloxacin | 0.664 | 0.358 | 2.386 | 0.017 | 0.170 |
| *Klebsiella* spp. | Trimethoprim-sulfamethoxazole | Amoxicillin-clavulanic acid | 0.664 | 0.475 | 1.467 | 0.142 | 1.000 |
| *Klebsiella* spp. | Nitrofurantoin | Ceftriaxone | 0.283 | 0.446 | -1.406 | 0.160 | 1.000 |
| *Klebsiella* spp. | Nitrofurantoin | Ciprofloxacin | 0.283 | 0.358 | -0.659 | 0.510 | 1.000 |
| *Klebsiella* spp. | Nitrofurantoin | Amoxicillin-clavulanic acid | 0.283 | 0.475 | -1.676 | 0.094 | 0.937 |
| *Klebsiella* spp. | Ceftriaxone | Ciprofloxacin | 0.446 | 0.358 | 0.773 | 0.440 | 1.000 |
| *Klebsiella* spp. | Ceftriaxone | Amoxicillin-clavulanic acid | 0.446 | 0.475 | -0.253 | 0.800 | 1.000 |
| *Klebsiella* spp. | Ciprofloxacin | Amoxicillin-clavulanic acid | 0.358 | 0.475 | -1.041 | 0.298 | 1.000 |

**Figure** **S1:** Forest plot showing the prevalence of *E. coli* resistance to trimethoprim-sulfamethoxazole. Individual study results are presented alongside the country and number of isolates analysed (n=) per country with 95% confidence intervals. The proportion of resistance is summarised (with 95% confidence intervals) for each 5-year time block (e.g. 2000-2004, 2005-2009, 2010-2014, 2015-2019, 2020-2025) and this is shown in blue.

**Figure S2:** Forest plot showing the prevalence of *E. coli* resistance to ciprofloxacin. Individual study results are presented alongside the country and number of isolates analysed (n=) per country with 95% confidence intervals. The proportion of resistance is summarised (with 95% confidence intervals) for each 5-year time block (e.g. 2000-2004, 2005-2009, 2010-2014, 2015-2019, 2020-2025) and this is shown in blue.

**Figure S3:** Forest plot showing the prevalence of *E. coli* resistance to ceftriaxone. Individual study results are presented alongside the country and number of isolates analysed (n=) per country with 95% confidence intervals. The proportion of resistance is summarised (with 95% confidence intervals) for each 5-year time block (e.g. 2000-2004, 2005-2009, 2010-2014, 2015-2019, 2020-2025) and this is shown in blue.

**Figure S4:** Forest plot showing the prevalence of *Klebsiella* spp*.* resistance to amoxicillin-clavulanic acid. Individual study results are presented alongside the country and number of isolates analysed (n=) per country with 95% confidence intervals (CI). The studies are ordered chronologically according to their study midpoint. The summary of amoxicillin-clavulanic acid resistance is shown in blue with 95% confidence intervals.

**Figure S5:** Forest plot showing the prevalence of *Klebsiella* spp*.* resistance to trimethoprim-sulfamethoxazole. Individual study results are presented alongside the country and number of isolates analysed (n=) per country with 95% confidence intervals (CI). The studies are ordered chronologically according to their study midpoint. The summary of trimethoprim-sulfamethoxazole resistance is shown in blue with 95% confidence intervals.

**Figure S6:** Forest plot showing the prevalence of *Klebsiella* spp*.* resistance to ciprofloxacin. Individual study results are presented alongside the country and number of isolates analysed (n=) per country with 95% confidence intervals (CI). The studies are ordered chronologically according to their study midpoint. The summary of ciprofloxacin resistance is shown in blue with 95% confidence intervals.

**Figure S7:** Forest plot showing the prevalence of *Klebsiella* spp*.* resistance to ceftriaxone. Individual study results are presented alongside the country and number of isolates analysed (n=) per country with 95% confidence intervals (CI). The studies are ordered chronologically according to their study midpoint. The summary of ceftriaxone resistance is shown in blue with 95% confidence intervals.

**Table S6:** Publication bias assessment. Table showing Egger’s test result for all organism-antibiotic combinations with over 10 studies

| **Organism** | **Antibiotic** | **Number of studies (isolates)** | **Pooled resistance prevalence (%)** | **95% Confidence Interval** | **I² statistic (%)** | **Egger’s Test p-value** |
| --- | --- | --- | --- | --- | --- | --- |
| *E. coli* | Trimethoprim-sulfamethoxazole | 22 (4888) | 62.2 | 51.9–72.5 | 99.27 | 0.9748 |
| *E. coli* | Nitrofurantoin | 24 (2656) | 20.3 | 10.4–30.2 | 99.31 | **0.0068** |
| *E. coli* | Ciprofloxacin | 31 (5602) | 32.8 | 25.1–40.4 | 98.76 | 0.0511 |
| *Klebsiella* spp. | Ciprofloxacin | 14 (907) | 35.8 | 20.3–51.4 | 98.65 | 0.7565 |
| *E. coli* | Amoxicillin-clavulanic acid | 30 (5898) | 48.9 | 39.8–58.0 | 98.53 | 0.2767 |
| *Klebsiella* spp. | Amoxicillin-clavulanic acid | 12 (768) | 47.5 | 31.8–63.2 | 97.68 | 0.7994 |
| *E. coli* | Ceftriaxone | 22 (3134) | 34.5 | 22.4–46.6 | 99.28 | 0.3619 |

**Figure S8:** Funnel plot of *E. coli* resistance to Nitrofurantoin before and after trim-and-fill analysis, where no changes were made.

**Table** **S7:** Subgroup analysis according to CLSI guideline changes

| **CLSI group** | **Organism** | **Antibiotic** | **No. Studies** | **No. Isolates** | **Pooled prevalence (95% CI)** | **I² statistic** | **QM statistic** | **p-value** |
| --- | --- | --- | --- | --- | --- | --- | --- | --- |
| Legacy (CLSI) | *E. coli* | Ciprofloxacin | 11 | 562 | 27.0 (18.0-36.0) | 85.814 | 2.244 | 0.134 |
| Modern/Standard |  |  | 14 | 3929 | 41.0 (28.0-54.0) | 98.522 |  |  |
| Legacy (CLSI) | *Klebsiella* spp*.* | Ciprofloxacin | 4 | 171 | 20.7 (7.4-33.9) | 76.611 | 2.155 | 0.142 |
| Modern/Standard |  |  | 8 | 371 | 47.7 (24.9-70.5) | 97.859 |  |  |
| Legacy (CLSI) | *E. coli* | Amoxicillin-clavulanic acid | 6 | 207 | 49.0 (25.4-72.6) | 94.142 | 0.126 | 0.723 |
| Modern/Standard |  |  | 18 | 4579 | 52.9 (42.3-63.4) | 98.343 |  |  |
| Legacy (CLSI) | *Klebsiella* spp*.* | Amoxicillin-clavulanic acid | 1 | 10 | 30.0 (1.6-58.4) | 0 | 0.519 | 0.471 |
| Modern/Standard |  |  | 9 | 393 | 53.5 (34.8-72.2) | 96.721 |  |  |
| Legacy (CLSI) | *E. coli* | Trimethoprim-sulfamethoxazole | 6 | 443 | 70.2 (54.0-86.3) | 95.549 | 0.005 | 0.943 |
| Modern/Standard |  |  | 10 | 3316 | 70.0 (60.6-79.4) | 96.534 |  |  |
| Legacy (CLSI) | *Klebsiella* spp*.* | Trimethoprim-sulfamethoxazole | 3 | 156 | 88.8 (67.4-110.1) | 98.304 | 0.933 | 0.334 |
| Modern/Standard |  |  | 4 | 88 | 67.9 (38.9-96.9) | 92.876 |  |  |
| Legacy (CLSI) | *E. coli* | Nitrofurantoin | 8 | 497 | 25.4 (10.2-40.6) | 96.392 | 0.964 | 0.326 |
| Modern/Standard |  |  | 10 | 1027 | 14.5 (-1.1-30.1) | 99.455 |  |  |
| Legacy (CLSI) | *Klebsiella* spp*.* | Nitrofurantoin | 3 | 156 | 29.4 (4.0-54.9) | 91.549 | 0.022 | 0.883 |
| Modern/Standard |  |  | 4 | 222 | 27.4 (-2.6-57.4) | 97.732 |  |  |
| Legacy (CLSI) | *E. coli* | Ceftriaxone | 7 | 276 | 38.4 (13.6-63.2) | 97.815 | 0.001 | 0.981 |
| Modern/Standard |  |  | 12 | 2024 | 38.0 (22.0-54.0) | 99.053 |  |  |
| Legacy (CLSI) | *Klebsiella* spp*.* | Ceftriaxone | 2 | 50 | 45.7 (25.4-65.9) | 50.102 | 0.185 | 0.667 |
| Modern/Standard |  |  | 6 | 318 | 53.3 (32.9-73.7) | 94.059 |  |  |
